# (*E*)-3-Amino-4-(2-phenyl­hydrazinyl­idene)-1*H*-pyrazol-5(4*H*)-one

**DOI:** 10.1107/S1600536812050854

**Published:** 2013-01-04

**Authors:** Galal H. Elgemeie, Shahinaz H. Sayed, Peter G. Jones

**Affiliations:** a Chemistry Department, Faculty of Science, Helwan University, Cairo, Egypt; b Institut für Anorganische und Analytische Chemie, Technische Universität Braunschweig, Postfach 3329, D-38023 Braunschweig, Germany

## Abstract

The mol­ecule of the title compound, C_9_H_9_N_5_O, is essentially planar (r.m.s. deviation of all atoms = 0.02 Å) except for the NH_2_ H atoms. An intra­molecular hydrazinyl­idene–carbonyl N—H⋯O=C hydrogen bond is present. In the crystal, mol­ecules are connected *via* N—H⋯N/O hydrogen bonds, forming thick layers parallel to (100).

## Related literature

The synthesis, chemistry and biological/medical activity of related compounds is described in: Elgemeie (2003 ▶); Elgemeie & El-Aziz (2002 ▶); Elgemeie & Sood (2003 ▶, 2006 ▶); Elgemeie *et al.* (2001 ▶, 2007 ▶, 2008 ▶, 2009 ▶).
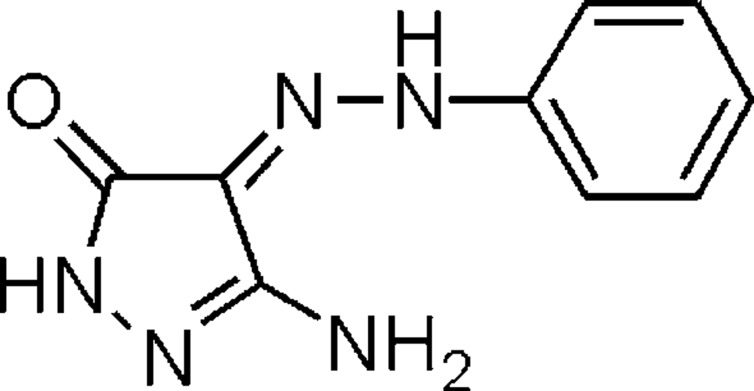


## Experimental

### 

#### Crystal data

C_9_H_9_N_5_O
*M* *_r_* = 203.21
Monoclinic, 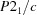

*a* = 6.7380 (2) Å
*b* = 13.4310 (4) Å
*c* = 10.4563 (3) Å
β = 103.094 (3)°
*V* = 921.67 (5) Å^3^
*Z* = 4
Cu *K*α radiation
μ = 0.86 mm^−1^
*T* = 100 K
0.15 × 0.10 × 0.03 mm

#### Data collection

Oxford Diffraction Xcalibur (Atlas, Nova) diffractometer
Absorption correction: multi-scan (*CrysAlis PRO*; Oxford Diffraction, 2009 ▶) *T*_min_ = 0.668, *T*_max_ = 1.000
26682 measured reflections
1914 independent reflections
1807 reflections with *I* > 2σ(*I*)
*R*_int_ = 0.029

#### Refinement

*R*[*F* ^2^ > 2σ(*F* ^2^)] = 0.032
*wR*(*F* ^2^) = 0.087
*S* = 1.05
1914 reflections
152 parameters
H atoms treated by a mixture of independent and constrained refinement
Δρ_max_ = 0.17 e Å^−3^
Δρ_min_ = −0.26 e Å^−3^

### 

Data collection: *CrysAlis PRO* (Oxford Diffraction, 2009 ▶); cell refinement: *CrysAlis PRO*; data reduction: *CrysAlis PRO*; program(s) used to solve structure: *SHELXS97* (Sheldrick, 2008 ▶); program(s) used to refine structure: *SHELXL97* (Sheldrick, 2008 ▶); molecular graphics: *XP* in *SHELXTL* (Sheldrick, 2008 ▶); software used to prepare material for publication: *SHELXL97*.

## Supplementary Material

Crystal structure: contains datablock(s) I, global. DOI: 10.1107/S1600536812050854/gg2105sup1.cif

Structure factors: contains datablock(s) I. DOI: 10.1107/S1600536812050854/gg2105Isup3.hkl

Supplementary material file. DOI: 10.1107/S1600536812050854/gg2105Isup3.cml

Additional supplementary materials:  crystallographic information; 3D view; checkCIF report

## Figures and Tables

**Table 1:** Hydrogen-bond geometry (Å, °)

*D*—H⋯*A*	*D*—H	H⋯*A*	*D*⋯*A*	*D*—H⋯*A*
N1—H01⋯O1^i^	0.909 (16)	1.949 (16)	2.8521 (11)	172.0 (14)
N3—H03*B*⋯N2^ii^	0.934 (17)	2.424 (16)	3.2711 (12)	150.8 (13)
N3—H03*A*⋯O1^iii^	0.908 (16)	2.141 (15)	2.9635 (11)	150.2 (13)
N5—H05⋯O1	0.897 (16)	2.174 (16)	2.8575 (11)	132.5 (13)

Symmetry codes: (i) 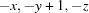; (ii) 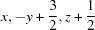; (iii) 
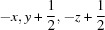
.

**Fig. 1.**
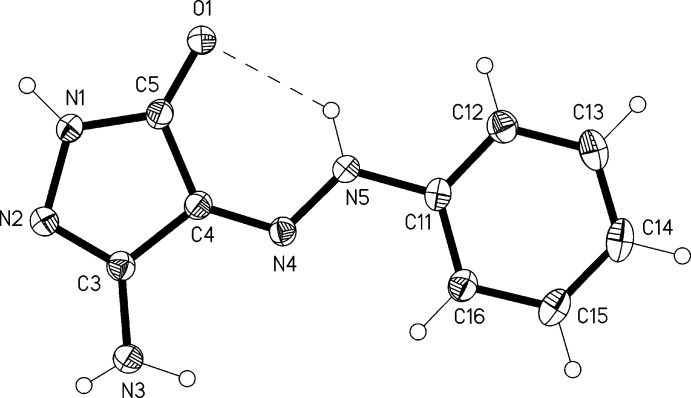
Molecular structure of the title compound. Ellipsoids represent 50% probability levels.

**Fig. 2.**
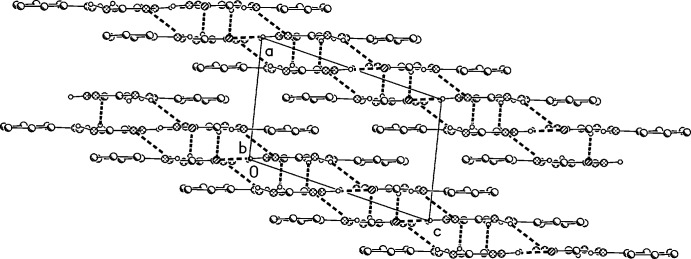
Packing diagram of the title compound projected along the b axis, showing the layer structure side-on.

**Fig. 3.**
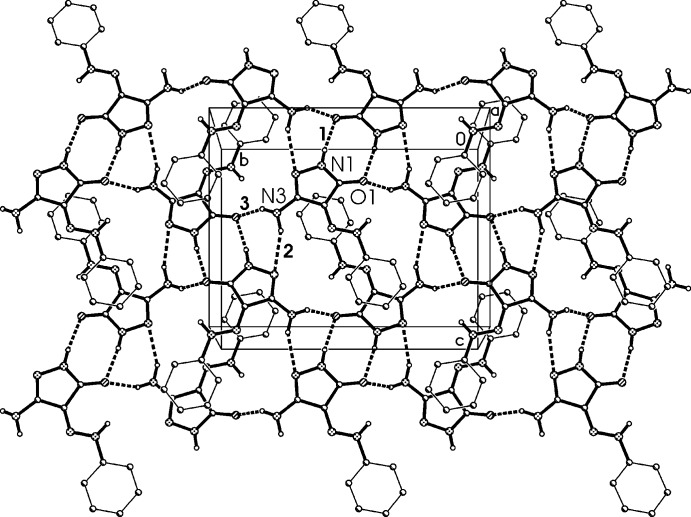
Packing diagram of the title compound, viewed perpendicular to (100). Thick dashed bonds represent classical H bonds. Atom names correspond to the asymmetric unit; hydrogen bonds are numbered according to the Table on page Sup-7 (#4, the weaker part of a three-centre interaction, is omitted, as is the intramolecular interaction #5).

**Fig. 4.**
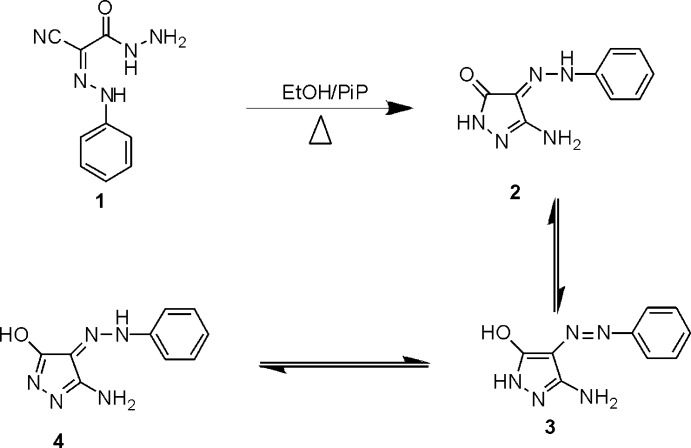
The formation of the title compound

## supplementary crystallographic information

### Comment

Chemically synthesized purine analogues find numerous applications in clinical medicine and medical research (Elgemeie, 2003; Elgemeie *et al.*, 2008). The pharmacological approach involves analogues in which the heterocyclic ring system has been modified so as to induce toxic effects when the analogue is incorporated into specific cell constituents (Elgemeie & El-Aziz, 2002). As part of our program directed towards the synthesis of purines and other antimetabolites (Elgemeie *et al.*, 2001, 2009), we have recently reported various successful approaches to the syntheses of purine analogues. Derivatives of these ring systems are of interest as antimetabolites in biochemical reactions (Elgemeie & Sood, 2003). We have described several novel syntheses of functionalized pyrazoles (Elgemeie *et al.*, 2007). These compounds are considered important intermediates for the synthesis of various purine ring systems (Elgemeie & Sood, 2006). As a continuation of this work, the title pyrazole compound (2), was prepared as a precursor for the synthesis of other purines. 2-Hydrazinyl-2-oxo-*N*-phenylacetohydrazonoyl cyanide (1) undergoes intramolecular cyclization by refluxing in ethanol containing catalytic amounts of piperidine to give the novel pyrazole derivative (2). The title compound can potentially exist in two other tautomeric forms with hydroxyl groups, (3) and (4). Spectral studies, however, indicated the presence of the ketonic tautomer (2) in solution (*e.g.* the ^13^C NMR signal at δ = 174.00, indicating a carbonyl carbon rather than C—OH.

The X-ray analysis of (2) (Fig. 1) establishes the exclusive presence of the keto tautomer in the solid state; all H atoms could be located unambiguously and bond lengths are also consistent with the keto form. The entire molecule is planar (r.m.s. deviation of all non-C atoms: 0.02 Å), except for the H atoms of the NH_2_ group; H03A lies 0.36 (2) and H03B 0.27 (2) Å outside the plane. Consistent with the *E* configuration, an intramolecular hydrogen bond N5—H05···O1 is observed.

The molecules are connected by hydrogen bonds #1–#3 to form thick hydrogen-bonded layers parallel to (100); the individual molecules are to a good approximation oriented in the planes (042) (Figs. 2, 3). Hydrogen bond #4 is the second and appreciably less linear branch of a three-centre interaction.

### Experimental

The title compound was obtained by refluxing an ethanolic solution of 2-hydrazinyl-2-oxo-*N*-phenylacetohydrazonoyl cyanide containing a few drops of piperidine for 1 h. After cooling, the precipitate was filtered off and recrystallized from ethanol. Yield (85%); m.p. 245 °C; IR (KBr) ν = 3450, 3350, 3300 (NH_2_, NH), 1660 (C═O, s) cm^-1^; ^1^H NMR (DMSO) δ = 6.88 (s, br, 2H, NH_2_), 7.23 (s, br, 1H, NH), 7.41–7.92 (m, 5H, C_6_H_5_); MS, m/*z* = 203; Calc. for C_9_H_9_N_5_O: C, 53.19; H, 4.46; N, 34.46; O, 7.87. Found: C, 53.56; H, 4.57; N, 34.62; O, 7.61%.

### Refinement

The NH H atoms were refined freely. Other H atoms were placed in calculated positions and refined using a riding model with C—H_arom_ 0.95 Å; the hydrogen *U* values were fixed at 1.2 × *U*(eq) of the parent atom.

### Crystal data

**Table:**

C_9_H_9_N_5_O	*F*(000) = 424
*M**_r_* = 203.21	*D*_x_ = 1.464 Mg m^−^^3^
Monoclinic, *P*2_1_/*c*	Melting point: 518 K
Hall symbol: -P 2ybc	Cu *K*α radiation, λ = 1.54184 Å
*a* = 6.7380 (2) Å	Cell parameters from 18413 reflections
*b* = 13.4310 (4) Å	θ = 3.3–75.6°
*c* = 10.4563 (3) Å	µ = 0.86 mm^−^^1^
β = 103.094 (3)°	*T* = 100 K
*V* = 921.67 (5) Å^3^	Tablet, orange-brown
*Z* = 4	0.15 × 0.10 × 0.03 mm

### Data collection

**Table:**

Oxford Diffraction Xcalibur (Atlas, Nova) diffractometer	1914 independent reflections
Radiation source: Nova (Cu) X-ray Source	1807 reflections with *I* > 2σ(*I*)
Mirror monochromator	*R*_int_ = 0.029
Detector resolution: 10.3543 pixels mm^-1^	θ_max_ = 75.8°, θ_min_ = 5.5°
ω–scan	*h* = −8→8
Absorption correction: multi-scan (*CrysAlis PRO*; Oxford Diffraction, 2009)	*k* = −16→16
*T*_min_ = 0.668, *T*_max_ = 1.000	*l* = −13→13
26682 measured reflections	

### Refinement

**Table:**

Refinement on *F*^2^	Primary atom site location: structure-invariant direct methods
Least-squares matrix: full	Secondary atom site location: difference Fourier map
*R*[*F*^2^ > 2σ(*F*^2^)] = 0.032	Hydrogen site location: inferred from neighbouring sites
*wR*(*F*^2^) = 0.087	H atoms treated by a mixture of independent and constrained refinement
*S* = 1.05	*w* = 1/[σ^2^(*F*_o_^2^) + (0.0473*P*)^2^ + 0.2869*P*] where *P* = (*F*_o_^2^ + 2*F*_c_^2^)/3
1914 reflections	(Δ/σ)_max_ < 0.001
152 parameters	Δρ_max_ = 0.17 e Å^−^^3^
0 restraints	Δρ_min_ = −0.26 e Å^−^^3^

### Special details

**Table:**

Geometry. All e.s.d.'s (except the e.s.d. in the dihedral angle between two l.s. planes) are estimated using the full covariance matrix. The cell e.s.d.'s are taken into account individually in the estimation of e.s.d.'s in distances, angles and torsion angles; correlations between e.s.d.'s in cell parameters are only used when they are defined by crystal symmetry. An approximate (isotropic) treatment of cell e.s.d.'s is used for estimating e.s.d.'s involving l.s. planes.Least-squares planes (*x*,*y*,*z* in crystal coordinates) and deviations from them (* indicates atom used to define plane)- 6.7047 (0.0004) *x* + 0.5009 (0.0024) *y* + 3.2946 (0.0013) *z* = 0.2057 (0.0017)* -0.0265 (0.0007) O1 * -0.0015 (0.0008) N1 * 0.0248 (0.0008) N2 * 0.0064 (0.0007) N3 * -0.0035 (0.0008) N4 * 0.0047 (0.0008) N5 * 0.0127 (0.0009) C3 * -0.0173 (0.0009) C4 * -0.0155 (0.0009) C5 * 0.0096 (0.0009) C11 * 0.0334 (0.0009) C12 * 0.0207 (0.0009) C13 * -0.0104 (0.0009) C14 * -0.0239 (0.0009) C15 * -0.0136 (0.0009) C16 0.3618 (0.0145) H03A 0.2676 (0.0152) H03BRms deviation of fitted atoms = 0.0175
Refinement. Refinement of *F*^2^ against ALL reflections. The weighted *R*-factor *wR* and goodness of fit *S* are based on *F*^2^, conventional *R*-factors *R* are based on *F*, with *F* set to zero for negative *F*^2^. The threshold expression of *F*^2^ > σ(*F*^2^) is used only for calculating *R*-factors(gt) *etc*. and is not relevant to the choice of reflections for refinement. *R*-factors based on *F*^2^ are statistically about twice as large as those based on *F*, and *R*- factors based on ALL data will be even larger.

### Fractional atomic coordinates and isotropic or equivalent isotropic displacement parameters (Å^2^)

**Table:**

	*x*	*y*	*z*	*U*_iso_*/*U*_eq_	
O1	0.09284 (11)	0.44649 (5)	0.17545 (7)	0.02234 (19)	
N1	0.06030 (14)	0.60858 (6)	0.09217 (8)	0.0210 (2)	
H01	0.008 (2)	0.5972 (11)	0.0053 (16)	0.036 (4)*	
N2	0.08679 (13)	0.70841 (6)	0.13887 (8)	0.0210 (2)	
C3	0.15061 (15)	0.70044 (7)	0.26628 (10)	0.0188 (2)	
C4	0.16773 (15)	0.59729 (7)	0.30772 (10)	0.0182 (2)	
C5	0.10365 (15)	0.53946 (8)	0.18664 (9)	0.0190 (2)	
N3	0.19896 (14)	0.77858 (7)	0.35089 (9)	0.0230 (2)	
H03B	0.202 (2)	0.7633 (12)	0.4385 (16)	0.041 (4)*	
H03A	0.135 (2)	0.8364 (12)	0.3206 (15)	0.036 (4)*	
N4	0.22316 (12)	0.56774 (6)	0.42917 (8)	0.0180 (2)	
N5	0.22648 (13)	0.47167 (6)	0.45303 (8)	0.0193 (2)	
H05	0.189 (2)	0.4280 (12)	0.3870 (15)	0.034 (4)*	
C11	0.28700 (14)	0.43692 (8)	0.58298 (10)	0.0190 (2)	
C12	0.28637 (16)	0.33470 (8)	0.60446 (11)	0.0229 (2)	
H12	0.2442	0.2901	0.5329	0.028*	
C13	0.34813 (16)	0.29858 (8)	0.73178 (11)	0.0268 (3)	
H13	0.3487	0.2289	0.7473	0.032*	
C14	0.40897 (16)	0.36360 (9)	0.83626 (11)	0.0283 (3)	
H14	0.4516	0.3386	0.9232	0.034*	
C15	0.40732 (17)	0.46548 (9)	0.81331 (10)	0.0271 (3)	
H15	0.4484	0.5099	0.8851	0.033*	
C16	0.34645 (16)	0.50330 (8)	0.68682 (10)	0.0223 (2)	
H16	0.3454	0.5730	0.6715	0.027*	

### Atomic displacement parameters (Å^2^)

**Table:**

	*U*^11^	*U*^22^	*U*^33^	*U*^12^	*U*^13^	*U*^23^
O1	0.0293 (4)	0.0182 (4)	0.0181 (4)	−0.0032 (3)	0.0024 (3)	−0.0003 (3)
N1	0.0273 (5)	0.0194 (4)	0.0149 (4)	−0.0021 (3)	0.0018 (3)	0.0002 (3)
N2	0.0249 (4)	0.0182 (4)	0.0193 (4)	−0.0005 (3)	0.0038 (3)	0.0006 (3)
C3	0.0188 (5)	0.0190 (5)	0.0189 (5)	0.0015 (4)	0.0045 (4)	0.0008 (4)
C4	0.0188 (5)	0.0189 (5)	0.0166 (5)	0.0006 (4)	0.0036 (4)	−0.0002 (4)
C5	0.0199 (5)	0.0205 (5)	0.0164 (5)	−0.0012 (4)	0.0038 (4)	0.0008 (4)
N3	0.0310 (5)	0.0173 (4)	0.0199 (4)	0.0032 (4)	0.0042 (4)	−0.0004 (3)
N4	0.0190 (4)	0.0179 (4)	0.0172 (4)	0.0019 (3)	0.0042 (3)	0.0012 (3)
N5	0.0237 (4)	0.0178 (4)	0.0155 (4)	0.0001 (3)	0.0028 (3)	−0.0002 (3)
C11	0.0170 (4)	0.0228 (5)	0.0170 (5)	0.0015 (4)	0.0039 (4)	0.0034 (4)
C12	0.0216 (5)	0.0225 (5)	0.0246 (5)	0.0002 (4)	0.0049 (4)	0.0016 (4)
C13	0.0229 (5)	0.0262 (6)	0.0316 (6)	0.0021 (4)	0.0071 (4)	0.0107 (4)
C14	0.0239 (5)	0.0384 (7)	0.0220 (5)	0.0025 (5)	0.0040 (4)	0.0111 (5)
C15	0.0272 (5)	0.0360 (6)	0.0175 (5)	−0.0002 (5)	0.0034 (4)	0.0008 (4)
C16	0.0238 (5)	0.0242 (5)	0.0188 (5)	0.0006 (4)	0.0045 (4)	0.0010 (4)

### Geometric parameters (Å, º)

**Table:**

O1—C5	1.2548 (13)	C13—C14	1.3861 (17)
N1—C5	1.3387 (13)	C14—C15	1.3890 (17)
N1—N2	1.4242 (12)	C15—C16	1.3891 (15)
N2—C3	1.3083 (13)	N1—H01	0.909 (16)
C3—N3	1.3637 (13)	N3—H03B	0.934 (17)
C3—C4	1.4484 (13)	N3—H03A	0.908 (16)
C4—N4	1.3019 (13)	N5—H05	0.897 (16)
C4—C5	1.4645 (13)	C12—H12	0.9500
N4—N5	1.3134 (12)	C13—H13	0.9500
N5—C11	1.4069 (13)	C14—H14	0.9500
C11—C12	1.3914 (15)	C15—H15	0.9500
C11—C16	1.3918 (15)	C16—H16	0.9500
C12—C13	1.3892 (15)		
			
C5—N1—N2	114.23 (8)	C14—C15—C16	120.92 (10)
C3—N2—N1	105.00 (8)	C15—C16—C11	118.64 (10)
N2—C3—N3	124.94 (9)	C5—N1—H01	126.2 (10)
N2—C3—C4	111.62 (9)	N2—N1—H01	119.4 (10)
N3—C3—C4	123.43 (9)	C3—N3—H03B	114.5 (10)
N4—C4—C3	124.69 (9)	C3—N3—H03A	114.0 (9)
N4—C4—C5	130.16 (9)	H03B—N3—H03A	115.7 (14)
C3—C4—C5	105.11 (8)	N4—N5—H05	120.4 (10)
O1—C5—N1	128.54 (9)	C11—N5—H05	119.7 (10)
O1—C5—C4	127.43 (9)	C13—C12—H12	120.4
N1—C5—C4	104.03 (9)	C11—C12—H12	120.4
C4—N4—N5	118.26 (9)	C14—C13—H13	119.8
N4—N5—C11	119.87 (8)	C12—C13—H13	119.8
C12—C11—C16	121.13 (9)	C13—C14—H14	120.2
C12—C11—N5	118.19 (9)	C15—C14—H14	120.2
C16—C11—N5	120.67 (9)	C14—C15—H15	119.5
C13—C12—C11	119.21 (10)	C16—C15—H15	119.5
C14—C13—C12	120.42 (10)	C15—C16—H16	120.7
C13—C14—C15	119.68 (10)	C11—C16—H16	120.7
			
C5—N1—N2—C3	0.65 (12)	C3—C4—N4—N5	178.06 (9)
N1—N2—C3—N3	178.81 (9)	C5—C4—N4—N5	0.52 (16)
N1—N2—C3—C4	−0.17 (11)	C4—N4—N5—C11	179.53 (9)
N2—C3—C4—N4	−178.34 (9)	N4—N5—C11—C12	179.30 (8)
N3—C3—C4—N4	2.66 (16)	N4—N5—C11—C16	−0.93 (14)
N2—C3—C4—C5	−0.29 (12)	C16—C11—C12—C13	−0.62 (15)
N3—C3—C4—C5	−179.29 (9)	N5—C11—C12—C13	179.15 (9)
N2—N1—C5—O1	179.27 (9)	C11—C12—C13—C14	0.23 (16)
N2—N1—C5—C4	−0.81 (11)	C12—C13—C14—C15	0.24 (16)
N4—C4—C5—O1	−1.54 (18)	C13—C14—C15—C16	−0.33 (17)
C3—C4—C5—O1	−179.44 (10)	C14—C15—C16—C11	−0.05 (16)
N4—C4—C5—N1	178.55 (10)	C12—C11—C16—C15	0.53 (15)
C3—C4—C5—N1	0.65 (10)	N5—C11—C16—C15	−179.24 (9)

### Hydrogen-bond geometry (Å, º)

**Table:**

*D*—H···*A*	*D*—H	H···*A*	*D*···*A*	*D*—H···*A*
N1—H01···O1^i^	0.909 (16)	1.949 (16)	2.8521 (11)	172.0 (14)
N3—H03*B*···N2^ii^	0.934 (17)	2.424 (16)	3.2711 (12)	150.8 (13)
N3—H03*A*···O1^iii^	0.908 (16)	2.141 (15)	2.9635 (11)	150.2 (13)
N3—H03*B*···N1^ii^	0.934 (17)	2.674 (16)	3.2562 (13)	121.1 (12)
N5—H05···O1	0.897 (16)	2.174 (16)	2.8575 (11)	132.5 (13)

Symmetry codes: (i) −*x*, −*y*+1, −*z*; (ii) *x*, −*y*+3/2, *z*+1/2; (iii) −*x*, *y*+1/2, −*z*+1/2.
