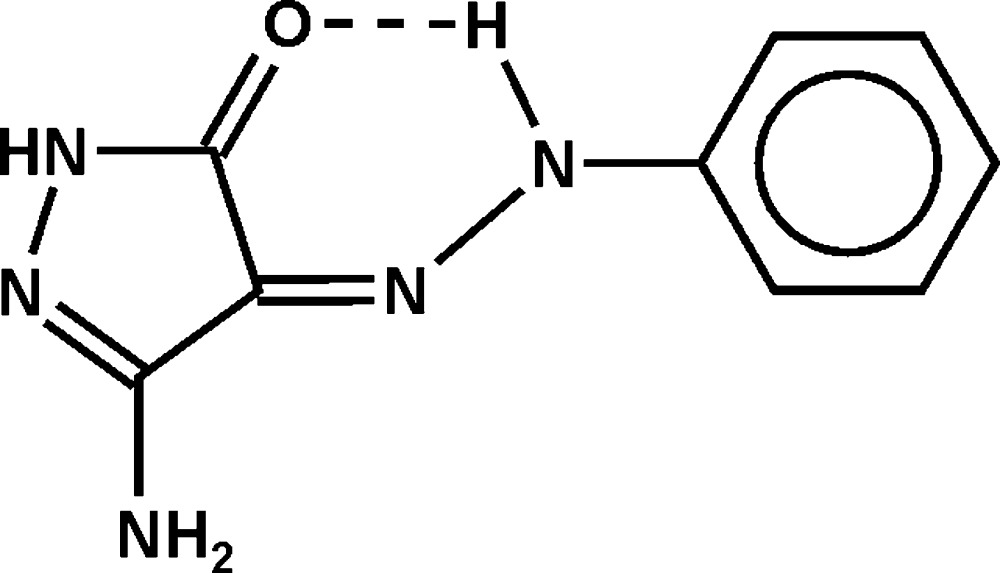# (*E*)-3-Amino-4-(2-phenyl­hydrazinyl­idene)-1*H*-pyrazol-5(4*H*)-one. Corrigendum

**DOI:** 10.1107/S160053681303403X

**Published:** 2013-12-21

**Authors:** Galal H. Elgemeie, Shahinaz H. Sayed, Peter G. Jones

**Affiliations:** a Chemistry Department, Faculty of Science, Helwan University, Cairo, Egypt; b Institut für Anorganische und Analytische Chemie, Technische Universität Braunschweig, Postfach 3329, D-38023 Braunschweig, Germany

## Abstract

The title in the paper by Elgemeie *et al.* [*Acta Cryst.* (2013), E**69**, o187] is corrected.

## Abstract 2

Corrigendum to *Acta Cryst.* (2013), E**69**, o187.

In the paper by Elgemeie *et al.* (2013) ▶, the scheme and title give the incorrect stereoisomer of the title compound. The correct title should be ‘(*Z*)-3-Amino-4-(2-phenyl­hydrazinyl­idene)-1*H*-pyrazol-5(4*H*)-one’ and the correct scheme is shown below.